# Supplementary figures and images for: mTORC1-mediated acquisition of reward-related representations by hippocampal somatostatin interneurons
Source: Mol Brain. 2023 Jul 3;16:55. doi: 10.1186/s13041-023-01042-w (PMC10318662; doi:10.1186/s13041-023-01042-w)

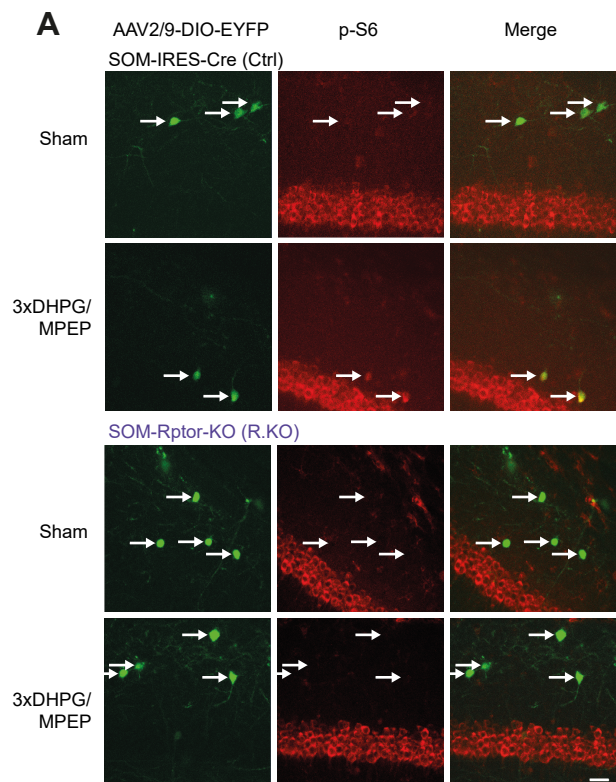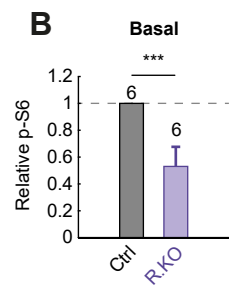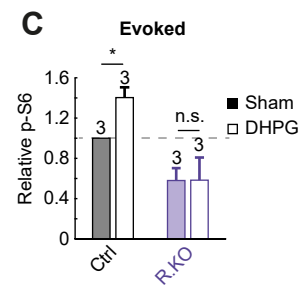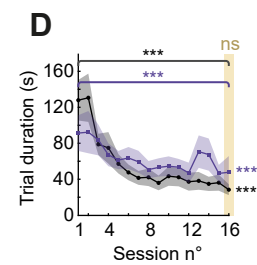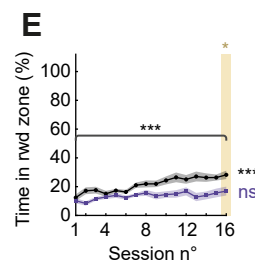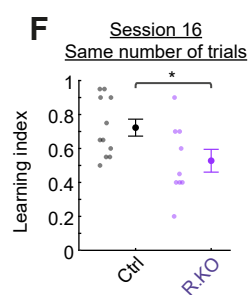

Supplement: Supplementary file 1 — Additional file 1: Figure S1. Inhibition of mTORC1 signaling in SOM-INs in SOM-Rptor-KO mice and other behavioral measures during learning. A Representative confocal immunofluorescence labelling of S6 phosphorylation in EYFP-expressing SOM-INs. Repeated mGluR1 stimulation increased p-S6 in SOM-INs relative to sham-treatment in slices from control SOM-IRES-Cre mice but not in slices from SOM-Rptor-KO mice. Scale bar: 20 µm. B Quantification of p-S6 immunofluorescence showing reduced basal level of p-S6 in SOM-INs of SOM-Rptor-KO mice relative to control SOM-IRES-Cre mice suggesting deficit of constitutive mTORC1 activity. C Quantification of evoked p-S6 showing increased p-S6 in SOM-INs after repeated mGluR1 stimulation relative to sham treatment of SOM-IRES-Cre mice but not in SOM-Rptor-KO mice confirming a deficit in mTORC1 signaling in SOM-INs of SOM-Rptor-KO mice. D, E Summary plots of changes over training sessions in SOM-IRES-Cre and SOM-Rptor-KO mice showing similar reduction in trial duration over training in both mice, and increase in percentage time spent in reward zone over training only in control SOM-IRES-Cre mice, indicative of a spatial learning deficit in SOM-Rptor-KO mice. F Summary plot of learning index measured for the same number of trials for each animal in the last training session, indicating a learning deficit in the SOM-Rptor-KO relative to control mice when trial numbers are constant. Details of statistical tests provided in Additional file 5: Table S1. * p < 0.05, *** p < 0.001, ns not significant. [file 13041_2023_1042_MOESM1_ESM.pdf]

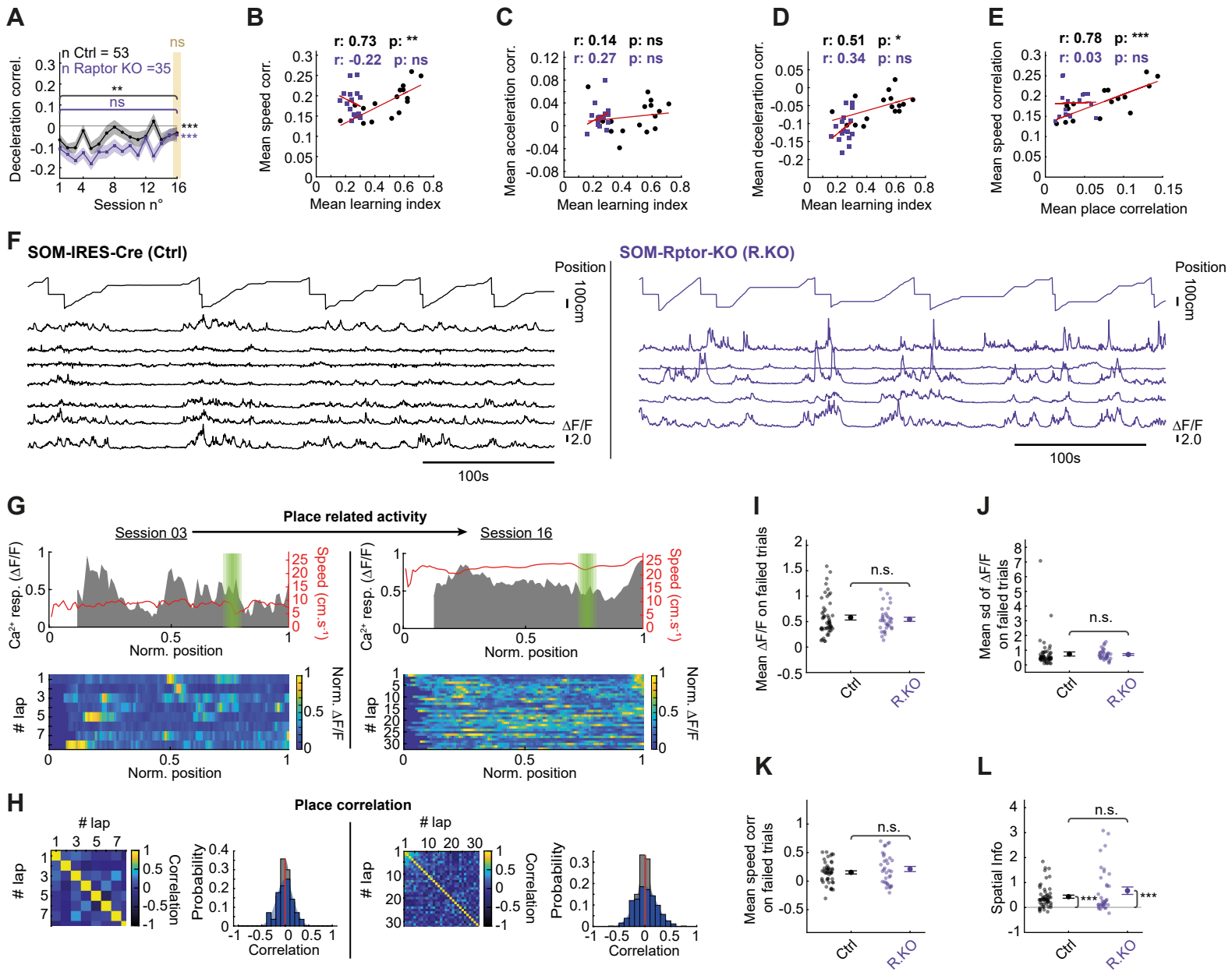

Supplement: Supplementary file 2 — Additional file 2: Figure S2. Deceleration correlation over training, and speed, acceleration, and deceleration correlations with mean learning index; and basal Ca2+ activity in SOM-Rptor-KO mice. A Mean correlation of Ca2+ activity with deceleration for all SOM-INs decreased at the end relative to the start of training in control but not in SOM-Rptor-KO mice. B Mean speed correlation as a function of mean learning index for all animals, showing correlation in control but not SOM-Rptor-KO mice. C Mean acceleration correlation as a function of mean learning index for all animals, showing absence of correlation. D Mean deceleration correlation as a function of mean learning index for all animals, showing correlation in control but not SOM-Rptor-KO mice. E Mean speed correlation as a function of mean place correlation for all animals, showing correlation in control but not SOM-Rptor-KO mice. F Examples of simultaneous measurements of position and Ca2+ responses during a training session from 7 SOM-INs of a control mouse and from 5 SOM-INs of a SOM-Rptor-KO mouse. G Ca2+ responses of a representative SOM-IN from a SOM-Rptor-KO mouse at start and end of training. Top: mean Ca2+ responsesand speed as function of position for all trials with reward zone indicated in green. Bottom: color-coded Ca2+ activity in each trial of the session, showing activity uncorrelated with position at the start and end of training. H Correlation of Ca2+ responses with position across laps at sessions 3 and 16, showing no place correlation at start and end of training. For each left: place correlation matrix of all paired laps. For each right: distribution of r values, mean r versus r distribution obtained by shuffling position measures. I-K Mean ΔF/F, mean standard deviation of ΔF/F and mean speed correlation in failed trials of the first 3 sessions of training, showing no difference between basal Ca2+ activity of SOM-INs of control and SOM-Rptor-KO mice during behavior-matched periods. L Mea [file 13041_2023_1042_MOESM2_ESM.pdf]

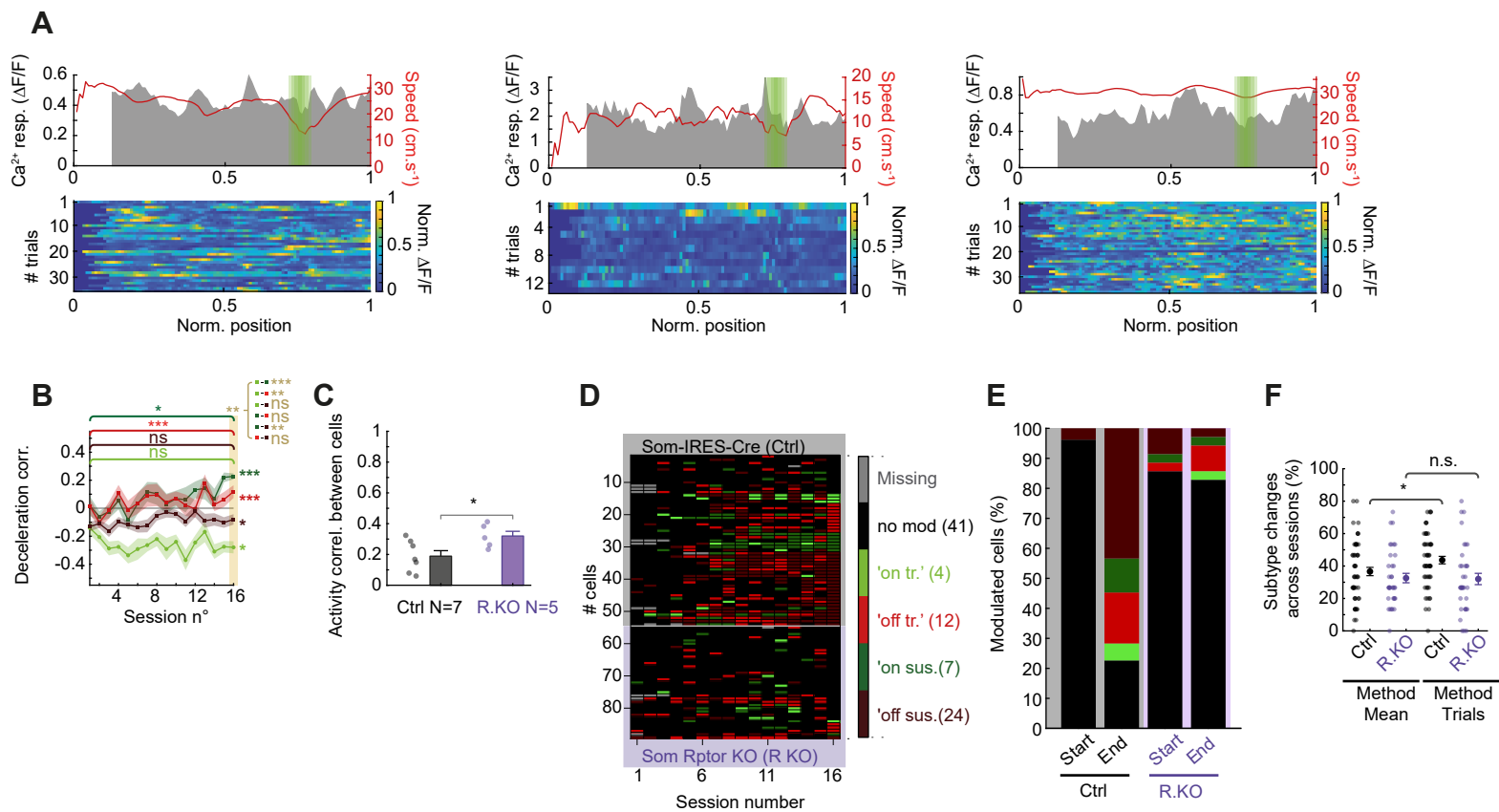

Supplement: Supplementary file 3 — Additional file 3: Figure S3. SOM-INs with no response modulation; deceleration correlation of response types; activity correlation between cells; and response type determined across trials. A Three representative examples of SOM-IN responses with no modulation related to reward. Top: mean Ca2+ responses and speed as function of position for all trials in a session with reward zone indicated in green. Bottom: color-coded Ca2+ activity in each trial of the session. B Similar representation as in Fig. 3F of Ca2+ activity correlation with deceleration for SOM-INs with different response types, showing response-specific changes over training. C Mean activity correlation between all SOM-INs, showing less correlation in control mice relative to SOM-Rptor-KO mice. D Cell response identity matrix obtained with a subtype identification method based on significant difference of ΔF/F across trials at reward zone. Response identity for all cells over training sessions ordered by response type at end of training, showing a gradual acquisition of spatial coding related to reward location. Top of matrix: SOM-INs from control mice. Bottom: SOM-INs from SOM-Rptor-KO mice. E Distribution of cells with different response types at start and end of training for control and SOM-Rptor-KO mice using subtype identification method based on trials, showing presence of 4 response types in both mouse genotypes, but increases with training in number of cells with responses only in control mice. F Summary plot for all cells comparing response subtype changes across all training sessions using response classification methods based on analysis of mean or trial activity, showing greater response variability with trial analysis method in control mice. Details of statistical tests provided in Additional file 5: Table S1. * p < 0.05, ** p < 0.01, *** p < 0.001, ns not significant. [file 13041_2023_1042_MOESM3_ESM.pdf]

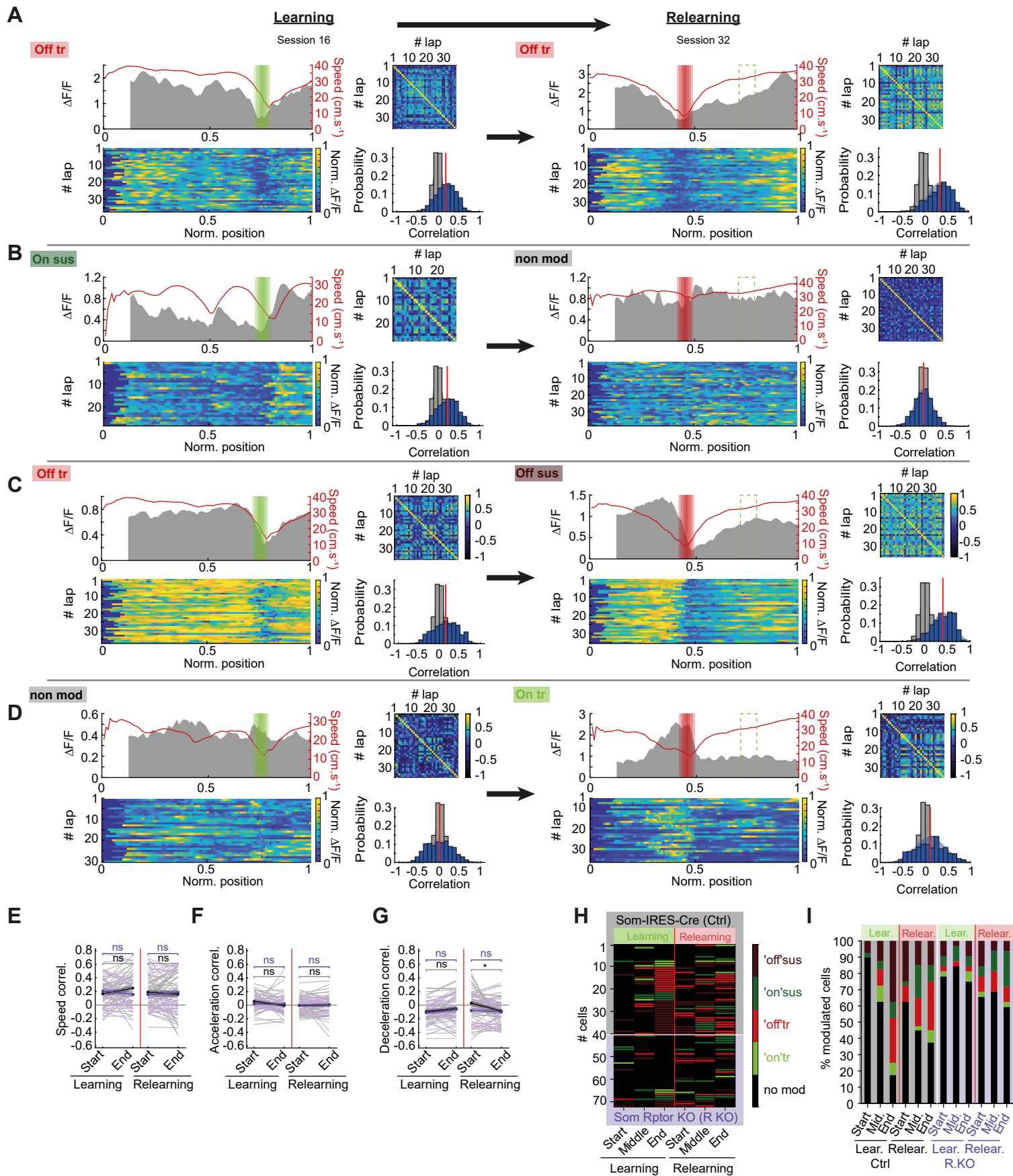

Supplement: Supplementary file 4 — Additional file 4: Figure S4. Examples of reorganization of SOM-IN activity, activity correlation, and response type distribution during relearning in control and SOM-Rptor-KO mice. A Example of no reorganization of SOM-IN responses for a cell with "reward off transient" responses at both end of learning and end of relearning. For each session, top left is mean Ca2+ responses and speed as function of position for all trials in the session with reward zone indicated; bottom left is color-coded Ca2+ activity in each trial of the session; top right is place correlation matrix of all paired laps; and bottom right is distribution of r values, mean r versus r distribution obtained by shuffling position measures. B Similar representation of reorganization for a SOM-IN with "reward on sustained" response at end of learning and "non-modulated" response at end of relearning. C Similar representation of reorganization for a SOM-IN with "reward off transient" response at end of learning and "reward off sustained" response at end of relearning. D Similar representation of reorganization for a SOM-IN with a "non-modulated" response at end of learning and "reward on transient" response at end of relearning. E Mean speed correlation with activity for all SOM-INs showing no change during relearning in control and SOM-Rptor-KO mice. F Mean acceleration correlation with activity for all SOM-INs showing no change during relearning in control and SOM-Rptor-KO mice. G Mean deceleration correlation with activity for all SOM-INs showing a decrease during relearning in control but not SOM-Rptor-KO mice. H Cell response identity matrix for all cells in control mice and SOM-Rptor-KO mice during learning and relearning ordered by response type at end of learning, showing a gradual acquisition of a new activity related to reward during relearning in control but not SOM-Rptor-KO mice. I Distribution of cells with different response types at start, middle and end of learning and relearning for co [file 13041_2023_1042_MOESM4_ESM.pdf]
